# Distinct CMR Phenotype in Alcoholic Cardiomyopathy: Greater Myocardial Fibrosis and Right Ventricular Dysfunction Compared with Idiopathic Dilated Cardiomyopathy

**DOI:** 10.3390/diagnostics16101560

**Published:** 2026-05-21

**Authors:** Víctor Vallejo-García, Manuel Barreiro-Pérez, David González-Calle, María del Carmen León del Pino, Victoria Jacas-Osborn, Carlos Barrios, Óscar Fabregat-Andrés

**Affiliations:** 1 Servicio de Cardiología, Hospital IMED Valencia, 46100 Burjassot, Spain; vvallejogarcia@gmail.com (V.V.-G.);; 2 Departamento de Medicina y Cirugía, Facultad de Ciencias de la Salud, Universidad Cardenal Herrera-CEU, CEU Universities, Alfara Del Patriarca, 46115 Valencia, Spain; 3 Servicio de Cardiología, Hospital Universitario Álvaro Cunqueiro, 36312 Vigo, Spain; 4 Servicio de Cardiología, Hospital Universitario de Salamanca, 37007 Salamanca, Spain; 5 Facultad de Medicina y Ciencias de la Salud, Universidad Católica de Valencia, 46001 Valencia, Spain; carlos.barrios@ucv.es

**Keywords:** alcoholic cardiomyopathy, cardiac magnetic resonance, late gadolinium enhancement, right ventricular dysfunction, dilated cardiomyopathy, myocardial fibrosis, biventricular function

## Abstract

**Background/Objectives**: Alcoholic cardiomyopathy (ACM) is a major preventable cause of non-ischemic dilated cardiomyopathy (DCM), yet its specific cardiac magnetic resonance (CMR) remains incompletely defined. We aimed to characterize the CMR features of ACM, focusing on late gadolinium enhancement (LGE) subpatterns and biventricular function and to compare them with idiopathic DCM. **Methods**: In total, 148 consecutive patients (ACM *n* = 20, idiopathic DCM n = 128) referred for CMR at a single center were retrospectively analyzed. Sequential logistic regression adjusted for age, sex, left ventricular ejection fraction (LVEF), and right ventricular ejection fraction (RVEF) was used to identify independent association with LGE presence. **Results**: LVEF did not differ between groups (32.5% vs. 35.0%, *p* = 0.293). ACM patients showed significantly worse RVEF (40.5% vs. 52.0%, *p* = 0.010) and larger indexed right ventricle (RV) volumes. Any LGE was present in 70% vs. 40% (*p* = 0.015); when the non-specific RV insertion point pattern (non-RV-IP) was excluded, non-RV-IP LGE was 45% vs. 22.7% (*p* = 0.051), with a specific midwall linear pattern (25% vs. 8%, *p* = 0.033). ACM was independently associated with LGE across all models with an adjusted odds ratio (OR) of 3.06 [95% CI 1.05–8.95], *p* = 0.041, and RV dysfunction (RVEF < 45%) (OR 4.79 [95% CI 1.60–14.32], *p* = 0.005). No differences in major adverse cardiovascular events (MACEs) were observed at 24 months (log-rank *p* = 0.697). **Conclusions**: ACM has a distinct CMR phenotype characterized by midwall linear LGE fibrosis and more severe RV involvement, independent of left ventricle (LV) systolic function. These exploratory findings suggest that CMR may provide clinically relevant phenotypic information in ACM beyond LVEF, warranting confirmation in prospective studies.

## 1. Introduction

Dilated cardiomyopathy (DCM) is characterized by progressive left ventricular dilatation and systolic dysfunction in the absence of significant coronary artery disease, significant valvular heart disease, or abnormal loading conditions, and it represents the most common indication for heart transplantation globally among young patients [1]. Among its etiologies, chronic excessive alcohol consumption accounts for 23–47% of DCM cases in recent European series that apply the recommended ESC guidelines’ intake thresholds (>80 g/day for ≥5 years), making alcoholic cardiomyopathy (ACM) one of the leading preventable forms of DCM in the world [1]. Unlike other causes of DCM, like idiopathic or genetic etiologies, ACM is partially reversible with heart failure medication and complete withdrawal of alcohol, with recovery of left ventricular ejection fraction (LVEF) and improvement of left ventricular volumes documented in a significant proportion of patients who achieve complete abstinence [1]. However, the myocardial fibrotic substrate does not completely recover, so there is an ongoing arrhythmic risk for ACM patients which has not been properly studied. There is sufficient evidence of the relationship between alcohol and atrial fibrillation, but there is a gap in knowledge regarding its association with ventricular arrhythmias and sudden cardiac death [2,3], and the presence of replacement fibrosis in ACM could be a potential trigger for ventricular arrhythmias and help to stratify the risk in patients.

Cardiac magnetic resonance (CMR) enables non-invasive myocardial tissue characterization through late gadolinium enhancement (LGE) and cine-derived biventricular volumetric assessment, making it the gold reference for the study of biventricular size and function and DCM phenotyping [4,5]. In idiopathic DCM, LGE identifies regions of replacement fibrosis in approximately 30–40% of patients, and it is an independent predictor of mortality and arrhythmic events after adjusting for LVEF [5,6]. Despite the growing role of CMR in DCM risk stratification, the specific CMR phenotype of ACM in direct comparison with idiopathic DCM remains incompletely defined. No prior study has simultaneously characterized and compared LGE subpatterns and biventricular function in ACM vs. idiopathic DCM using multivariable-adjusted analysis.

The objectives of our study were to characterize the CMR phenotype of ACM in direct comparison with idiopathic DCM from the same institutional registry, with specific focus on LGE subpatterns and biventricular function, and to assess the independent association of ACM etiology with LGE presence using a sequential logistic regression adjusted model.

## 2. Materials and Methods

### 2.1. Study Population

We retrospectively analyzed 148 consecutive patients with non-ischemic DCM referred for CMR at a single center between 2014 and 2019. Of these, 20 were classified as having ACM (chronic alcohol intake >80 g/day for ≥5 years, confirmed by standardized clinical interview) and 128 as having idiopathic DCM. Coronary artery disease was excluded in all patients by coronary angiography (Philips Allura Xper FD20, Philips Healthcare, Best, The Netherlands) or equivalent non-invasive testing coronary CT angiography (Philips iCT Brilliance 256 slices, Phillips Healthcare, Best, The Netherlands). Patients with ischemic cardiomyopathy, hypertrophic cardiomyopathy, cardiac amyloidosis, sarcoidosis, myocarditis, significant valvular heart disease, or peripartum cardiomyopathy were excluded. To assess the potential confounding effect of chronic obstructive pulmonary disease (COPD) on right ventricular parameters, a pre-specified sensitivity analysis excluding COPD patients was performed.

### 2.2. Cardiac Magnetic Resonance Protocol

**Figure 1:**
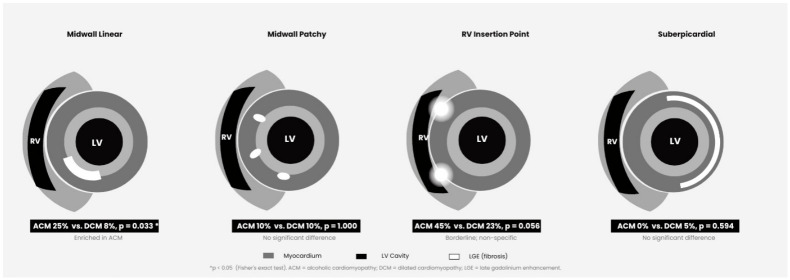
Schematic illustration of the four late gadolinium enhancement (LGE) patterns assessed in this study, shown in short-axis cardiac cross-section. Each panel shows the left ventricular (LV) myocardium (grey), cavity (black), right ventricle (RV, crescent), and LGE distribution (white). Midwall linear LGE (ACM 25% vs. DCM 8%, *p* = 0.033) was the only pattern which was significantly increased in ACM. RV insertion point LGE (ACM 45% vs. DCM 23%, *p* = 0.056) is a recognized non-specific finding that occurs in healthy individuals and carries no established prognostic significance. * *p*  <  0.05; (Fisher’s exact test). ACM, alcoholic cardiomyopathy; DCM, dilated cardiomyopathy; LGE, late gadolinium enhancement; LV, left ventricle; RV, right ventricle.

CMR was performed on 1.5T systems (Achieva DS, Philips Healthcare, Best, The Netherlands) following a standardized institutional protocol. Biventricular ejection fractions and indexed volumes were derived from standard steady-state free precession (SSFP) cine sequences in short-axis orientation. LGE was assessed on inversion-recovery gradient-echo sequences acquired 10–15 min after intravenous gadolinium administration (0.2 mmol/kg). LGE patterns (midwall linear, midwall patchy, RV insertion point, subepicardial), as illustrated in Figure 1, were classified by two experienced readers (both with more than 5 years of clinical experience and with the European Society of Cardiology Certification in Cardiovascular Magnetic Resonance) blinded to clinical and etiology data. In cases of disagreement, consensus was reached by discussion with a third reader.

### 2.3. Clinical Variables and Follow-Up

Demographic data, cardiovascular risk factors, NYHA functional class, medication use, and ECG findings were recorded at the time of CMR. Outcome data (major adverse cardiovascular events (MACEs): composite of all-cause death, heart failure hospitalization, or arrhythmia) were collected prospectively during clinical follow-up by the lead investigator.

### 2.4. Statistical Analysis

Categorical variables are reported as n (%). Continuous variables were tested for normality using the Shapiro–Wilk test. Non-normally distributed continuous variables are reported as medians (interquartile range, IQR) and compared using the Mann–Whitney U test. Categorical variables were compared using Fisher’s exact test or chi-squared test as appropriate. Survival analysis was performed using Kaplan–Meier curves with log-rank testing for comparison of MACE-free survival between groups.

Independent predictors of LGE presence were assessed by sequential logistic regression. Additionally, a separate multivariable logistic regression model was constructed with RV dysfunction (defined as RVEF < 45%, the threshold recommended in the 2023 ESC Guidelines for cardiomyopathies [4]) as a binary outcome, adjusting for age, sex, and LVEF, to formally test the independence of RV involvement from LV systolic impairment. A pre-specified sensitivity analysis excluding patients with COPD was performed for the RV dysfunction endpoint to assess the potential confounding effect of COPD on RV function. We conducted a pre-specified sensitivity analysis for LGE additionally adjusted for current smoking to address residual confounding. Four models were constructed: (1) unadjusted; (2) adjusted for age and sex; (3) adjusted for age, sex, and LVEF; and (4) full model adjusted for age, sex, LVEF, and RVEF. Continuous covariates were standardized (mean 0, SD 1). The events-per-variable ratio (EPV) was calculated as the number of outcome events divided by the number of covariates. EPV for the primary full model (Model 4, fully adjusted for covariates) was 13.0, exceeding the recommended threshold of 10. Post hoc power calculations using the two-proportion comparison confirmed 73% statistical power to detect the observed difference in LGE prevalence (70% vs. 40%) at α = 0.05 (two-sided), given the study sample sizes (ACM n = 20, DCM n = 128). For the RV dysfunction outcome (RVEF < 45%), post hoc power was more robust at 87% (60% vs. 24%), confirming adequate power for both primary endpoints despite the modest ACM sample size. Model calibration was assessed by the Hosmer–Lemeshow test. All statistical analyses were performed with Stata v16 (StataCorp, College Station, TX, USA). A two-sided *p*-value < 0.05 was considered statistically significant. To further assess the robustness of the primary result given the small ACM sample (n = 20), a non-parametric bootstrap was performed (1000 resamples with replacement). Of 998 valid iterations, the bootstrap percentile 95% CI for the ACM OR was 1.173–12.250 and P (OR  >  1.0) = 99.1%, supporting the directional robustness of the primary result. No generative artificial intelligence (GenAI) tools or large language models (LLMs) were used in this study, including data collection, manuscript preparation, statistical analysis, interpretation of results and figure preparation.

## 3. Results

### 3.1. Baseline Characteristics

The study population comprised 148 patients with a median age of 59.5 (IQR 52.0–69.8) years in the ACM group and 65.0 (IQR 51.8–73.0) years in the idiopathic DCM group (*p* = 0.558). The ACM group had a higher proportion of males (95% vs. 70%, *p* = 0.015) and current smokers (55% vs. 24%, *p* = 0.007) and a lower rate of familial cardiomyopathy (5% vs. 26%, *p* = 0.045). NYHA class ≥ III was present in 25% of ACM vs. 20% of idiopathic DCM patients (*p* = 0.570). Prior atrial fibrillation did not differ between groups (25% vs. 23%, *p* = 1.000). COPD was present in 4/20 ACM patients (20%) and 8/128 idiopathic DCM patients (6.3%; *p* = 0.059). No patient had known pulmonary fibrosis, chronic thromboembolic pulmonary hypertension, or other significant pulmonary pathologies beyond COPD. Full baseline characteristics, CMR parameters, and outcomes are presented in Table 1.

### 3.2. CMR Findings: Lv and Rv Function

LV function and volumes did not differ significantly between groups. Median LVEF was 32.5% (IQR 27.5–38.2) in ACM vs. 35.0% (IQR 26.8–44.0) in idiopathic DCM (*p* = 0.293), and indexed LV end-diastolic and end-systolic volumes were equally comparable (all *p* > 0.29).

**Figure 2:**
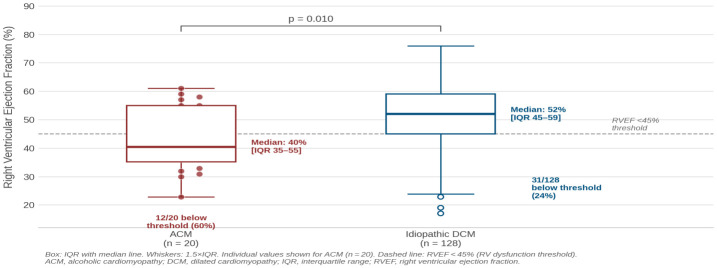
Individual right ventricular ejection fraction (RVEF) values by etiology. Box plot with individual patient values shown for ACM (n = 20, maroon) and idiopathic DCM (n = 128, navy). Boxes represent the interquartile range (IQR) with the median line. The dashed line indicates the RVEF  <  45% threshold for right ventricular dysfunction according to ESC guidelines. Note the heterogeneous RVEF distribution in ACM (IQR 35.2–55.0%) relative to the idiopathic DCM group. Mann–Whitney U, *p* = 0.010.

In contrast, ACM patients showed significantly impaired RV function and larger RV volumes (Figure 2). Median RVEF was 40.5% (IQR 35.2–55.0) vs. 52.0% (IQR 45.0–59.0) in the idiopathic DCM group (*p* = 0.010). Indexed RV end-diastolic volume was 96.0 (IQR 82.8–112.0) vs. 79.0 (IQR 68.5–95.0) mL/m^2^ (*p* = 0.004), and indexed RV end-systolic volume was 46.8 (IQR 40.8–69.8) vs. 36.0 (IQR 28.0–46.2) mL/m^2^ (*p* = 0.001).

**Figure 3:**
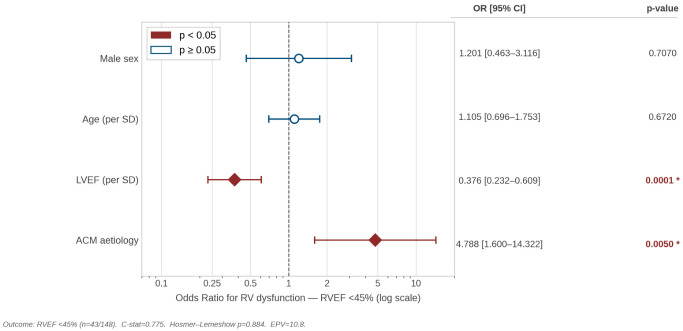
Multivariable predictors of right ventricular dysfunction (RVEF  <  45%). Forest plot of OR for all covariates in the fully adjusted logistic regression model (outcome: RVEF  <  45%; n = 148; events n = 43). ACM etiology and LVEF were independently associated with RV dysfunction. C-statistic = 0.775; Hosmer–Lemeshow test *p* = 0.884; EPV = 10.8. Filled diamonds: *p*  <  0.05; open circles: *p*  ≥  0.05. The asterisk means the results achieve statistical significance.

To formally test whether ACM etiology was independently associated with RV dysfunction, we constructed a multivariable logistic regression model with RVEF < 45% as the binary outcome, adjusting for age, sex, and LVEF. RVEF  <  45% was present in 12/20 ACM patients (60%) vs. 31/128 (24%) idiopathic DCM patients (Fisher’s exact *p* = 0.002), consistent with the significantly lower median RVEF in ACM (40.5% vs. 52.0%, Mann–Whitney *p* = 0.010; Table 1). In the adjusted model, ACM etiology was independently associated with RV dysfunction (OR 4.79 (95% CI 1.60–14.32), *p* = 0.005), while LVEF was the only other significant covariate (OR 0.38 per SD, 95% CI 0.23–0.61, *p* < 0.001), suggesting that RV involvement in ACM is not fully explained by LV systolic impairment alone (Figure 3). The model showed good discrimination (C-statistic 0.775) and adequate calibration (Hosmer–Lemeshow test *p* = 0.884). Events-per-variable (EPV) for this adjusted model was 10.8, meeting the recommended threshold. In a pre-specified sensitivity analysis excluding the 12 patients with COPD (ACM n = 16, DCM n = 120), RV dysfunction (RVEF < 45%) remained significantly more prevalent in ACM (10/16, 62.5% vs. 28/120, 23.3%; Fisher’s exact *p* = 0.002), and median RVEF remained significantly lower in ACM (38.5% vs. 52.0%; Mann–Whitney *p* = 0.010), suggesting that the RV dysfunction finding is not attributable to the higher COPD prevalence in the ACM group.

### 3.3. CMR Findings: Late Gadolinium Enhancement

**Figure 4:**
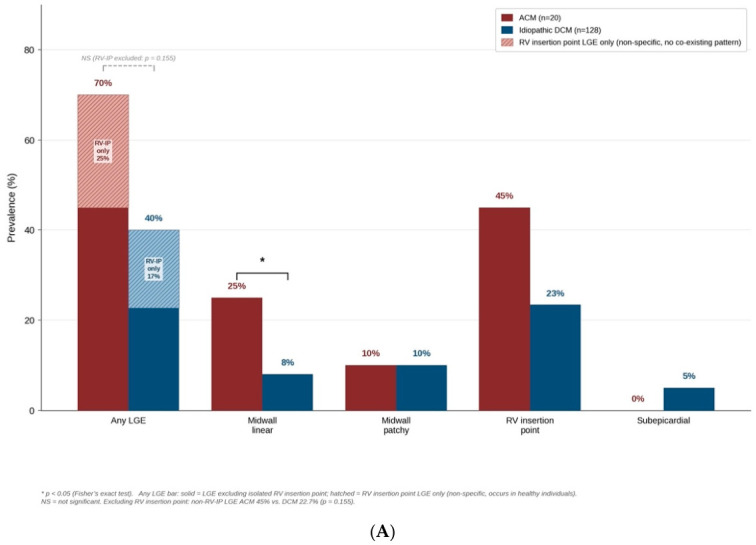

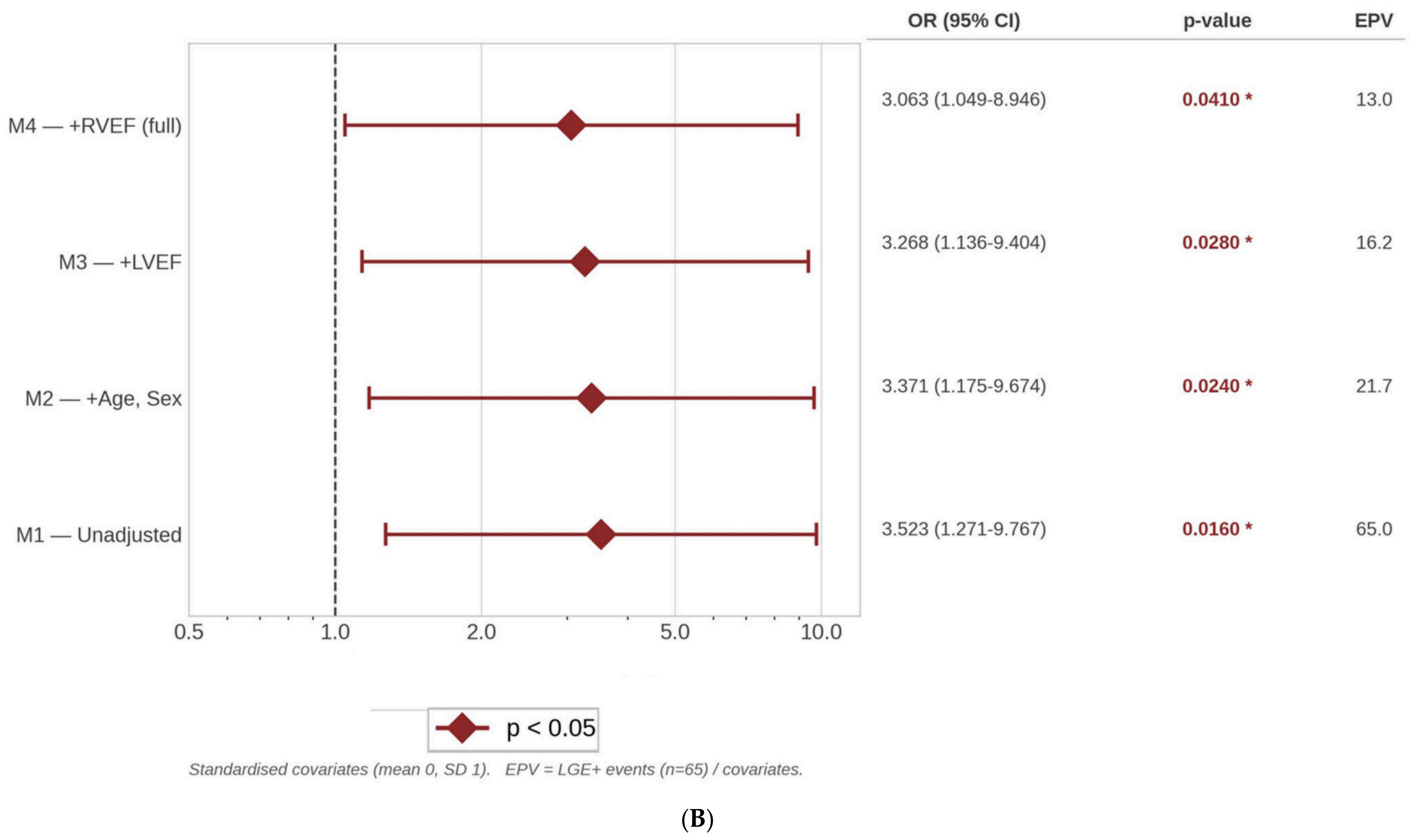
CMR characterization of alcoholic vs. idiopathic dilated cardiomyopathy. (**A**) Prevalence of LGE patterns in ACM (maroon) vs. idiopathic DCM (navy). The “Any LGE” bar is split: solid segments are patients with LGE excluding isolated RV insertion point; shaded segments are patients with RV insertion point LGE (non-specific, occurs in healthy individuals). When the RV insertion point pattern was excluded, non-RV-IP LGE: ACM 45% vs. DCM 22.7% (*p* = 0.051, NS). Significance markers: * *p* < 0.05 (Fisher’s exact test). (**B**) Forest plot of odds ratios (ORs) for LGE presence across sequential logistic regression models. Filled diamonds, *p* < 0.05. Dashed vertical line = OR 1.0. Continuous predictors standardized (per 1 SD). CI, confidence interval; LVEF, left ventricular ejection fraction; OR, odds ratio; RVEF, right ventricular ejection fraction; EPV, events-per-variable. The asterisk means the results achieve statistical significance (*p* < 0.05).

Any LGE was present in 14/20 (70%) ACM patients vs. 51/128 (40%) idiopathic DCM patients (*p* = 0.015). Given that the RV insertion point (RV-IP) LGE pattern is a non-specific finding that may occur in healthy individuals and carries no established prognostic significance, we conducted a pre specified sensitivity analysis of LGE excluding this pattern. When the RV-IP pattern was excluded, 9/20 ACM patients (45%) had LGE excluding the isolated RV-IP pattern, compared to 29/128 (22.7%) idiopathic DCM patients (*p* = 0.051, borderline non-significant but with a trend to a higher fibrosis burden in the ACM group). Among patients with any LGE, 5/20 ACM patients (25%) and 22/128 DCM patients (17.2%) had RV-IP as their sole LGE pattern, without other co-existing patterns. LGE pattern analysis revealed that the midwall linear pattern was specifically enriched in ACM (5/20 (25%) vs. 10/128 (8%), *p* = 0.033; Figure 4A). Midwall patchy (10% vs. 10%, *p* = 1.000), RV insertion point (45% vs. 23%, *p* = 0.056), and subepicardial (0% vs. 5%, *p* = 0.594) patterns did not differ significantly between groups.

On sequential logistic regression, ACM etiology was independently associated with LGE presence across all adjustment models (Figure 4B): unadjusted OR 3.52 (95% CI 1.27–9.77), *p* = 0.016; adjusted for age and sex: OR 3.37 (95% CI 1.17–9.67), *p* = 0.024; adjusted for age, sex, and LVEF: OR 3.27 (95% CI 1.14–9.40), *p* = 0.028; full model (age, sex, LVEF, RVEF): OR 3.06 (95% CI 1.05–8.95), *p* = 0.041; EPV = 13.0. Age was the only other significant covariate in the adjusted models (OR 1.59 per SD, *p* = 0.024). The Hosmer–Lemeshow test confirmed adequate model calibration (*p* = 0.214). ACM patients also had a higher overall LGE burden, with a greater median number of co-existing LGE patterns per patient (1 (IQR 0–1) vs. 0 (IQR 0–1), *p* = 0.044). In a pre-specified sensitivity analysis additionally adjusting for current smoking, the main confounding variable differing between groups, ACM etiology remained independently associated with LGE with an OR of 2.83 (95% CI 0.95–8.43, *p* = 0.062), while smoking itself was not independently associated (OR 1.36, 95% CI 0.60–3.09), *p* = 0.466). The attenuation of the ACM effect in this model is attributable to loss of statistical power due to limited sample size rather than true collinearity (VIF < 2.0 for all covariates), supporting ACM as the primary driver of LGE excess in this cohort of patients.

### 3.4. Clinical Outcomes

At a median follow-up of 24 months, MACE rates did not differ between ACM and idiopathic DCM patients (35% vs. 29%, log-rank *p* = 0.697). All-cause death occurred in 2/20 (10%) ACM vs. 11/128 (9%) idiopathic DCM patients (*p* = 0.689), and heart failure hospitalization in 4/20 (20%) vs. 17/128 (13%, *p* = 0.489).

## 4. Discussion

This study suggests that ACM has a distinct CMR phenotype relative to idiopathic DCM. Patients with ACM presented greater myocardial fibrosis with an adjusted OR of 3.06, 95% CI 1.05–8.95 for LGE presence, with specific enrichment of the midwall linear pattern, and more impaired RV function with larger RV volumes. LV function and LV volumes did not differ significantly between groups.

The pathophysiological basis for the excess LGE in ACM is well established in the available medical literature. Direct ethanol and acetaldehyde toxicity promotes focal cardiomyocyte apoptosis and necrosis, leading to laminar replacement fibrosis [1], which is assessed in the CMR with LGE sequences, like the midwall linear pattern that is specifically enriched in our ACM cohort. Importantly, all ACM patients in our cohort were actively consuming alcohol at the time of CMR, so the observed LGE burden, therefore, reflects the active fibrotic phenotype rather than residual post-abstinence remodeling. The midwall linear pattern has an independent arrhythmic risk in non-ischemic DCM. Halliday et al. [7] reported a hazard ratio (HR) of 9.2 for the composite of sudden cardiac death and aborted SCD associated with midwall LGE in patients with LVEF ≥40%, potentially contributing to the persistent arrhythmic risk in ACM even after LVEF recovery due to alcohol abstinence and optimal heart failure treatment. These findings have direct implications for ICD decision-making in ACM patients who recover LV function after abstinence but retain a significant fibrotic substrate. Our findings are exploratory due to the retrospective single-center nature of the research, but if they are confirmed in independent studies, the presence of a midwall linear LGE pattern in ACM could help to stratify and select high-risk patients for closer follow-up or even serve as an additional factor while assessing possible use of ICD if LVEF is sufficiently impaired.

The finding of significantly worse RV function in ACM, independent of LVEF after multivariable adjustment, is a novel finding, but it is biologically plausible and extends prior observations and case series. In idiopathic DCM, Gulati et al. [6] demonstrated that CMR-assessed RV systolic dysfunction (RVEF ≤ 45%) was present in 34% of 250 consecutive DCM patients and was a powerful independent predictor of transplant-free survival (HR 5.90, 95% CI 3.35–10.37), establishing RV ejection fraction as a clinically and statistically relevant prognostic parameter in non-ischemic DCM. Wang et al. [8] also documented biventricular impairment through ventricular interdependence specifically in ACM. Our multivariable analysis extends these observations: ACM etiology was the strongest predictor of RV dysfunction (OR 4.79, 95% CI 1.60–14.32, *p*= 0.005) after adjustment for age, sex, and LVEF, with LVEF itself also being a significant covariate (OR 0.38 per SD, *p* < 0.001). The persistence of the ACM effect independent of LV function argues against a purely secondary hemodynamic mechanism (contrary to Wang’s findings that suggested the mechanism was due to ventricular interdependence) and is more consistent with direct right ventricular toxicity of ethanol and its metabolites, as proposed in other papers assessing the physiologic effect of alcohol in the heart [1]. Notably, the RVEF distribution in the ACM group was heterogeneous in our sample (IQR 35.2–55.0%), with 8/20 patients (40%) maintaining RVEF ≥45%: this variability of phenotype may reflect differences in cumulative alcohol exposure, individual susceptibility, or disease stage at time of CMR acquisition. Given the small sample size and exploratory design of this study, these findings on RV function should be regarded as hypothesis-generating and require validation in larger prospective cohorts before any clinical implications can be drawn.

Artico et al. [9] reported replacement fibrosis measured by LGE in more than 40% of 52 ACM patients but did not perform multivariable adjustment for right ventricular (RV) function or analyze LGE subpatterns independently. Li et al. [10] confirmed the prognostic value of LGE in 141 ACM patients using DCM as a control group but did not assess RV function or LGE subpatterns. Our study addresses these issues directly. Compared to Artico et al. [9], which has one of the largest cohorts of ACM patients published, we corroborate and extend their findings by demonstrating that the LGE excess in ACM is independently maintained after multivariable adjustment for RV function and that the midwall linear subpattern specifically differentiates ACM from idiopathic DCM. Gulati et al. [11] demonstrated in a large prospective DCM cohort that midwall LGE was independently associated with all-cause mortality (HR 2.43, 95% CI 1.50–3.92) and arrhythmic events (HR 4.61, 95% CI 2.75–7.74), identifying this LGE pattern as a particularly malignant substrate. Our finding of a 70% LGE prevalence in ACM substantially exceeds the 30–40% typically reported in idiopathic DCM [5,11] and raises the hypothesis that ACM may represent a phenotype that is particularly prone to fibrosis replacement due to the toxic effects of ethanol and its metabolites, even if the prognostic implications of this fibrotic burden in the specific context of ACM remain to be established prospectively in independent studies. Even excluding the RV insertion point pattern, the presence of LGE remains higher than in the published literature (45%), and approaches statistical significance (*p* = 0.051), although this should be corroborated in other studies due to the small sample size. The borderline enrichment of the RV insertion point LGE pattern in ACM (45% vs. 23%, *p* = 0.056) did not reach statistical significance and should be interpreted cautiously given the small ACM sample; nevertheless, the biological plausibility is supported by the recognized hemodynamic sensitivity of the interventricular junction in states of sustained volume and pressure overload [8], and this observation warrants evaluation in larger cohorts. The absence of MACE differences at 24 months most likely reflects the limited follow-up duration and the small ACM sample size rather than a true absence of divergence of prognosis. Taken together, our results are exploratory and hypothesis-generating: adequately powered prospective studies with extended observation beyond 24 months and serial CMR to check for reverse remodeling are needed to determine whether the excess LGE and RV dysfunction that we observed in our cohort of actively drinking ACM patients translate into differential long-term outcomes relative to idiopathic DCM.

From a clinical perspective, the present findings should be interpreted in the context of what is published regarding the natural history of ACM. Amor-Salamanca et al. [12]. followed 101 ACM patients over a median of 82 months and found that 42% achieved significant LVEF recovery, which was associated with better outcomes (cardiovascular death or transplant 1% vs. 30%; *p* <  0.001), while none of the six patients who persisted with heavy alcohol use recovered LVEF. The updated review by Domínguez et al. [1] further confirms that complete abstinence from alcohol is critical, accentuating that the relationship between alcohol and arrhythmic risk in ACM is significant even in a contemporary treated cohort. In this study, LVEF recovery with abstinence and heart failure treatment is associated with a better prognosis, but there remains a subset of patients with ACM and maintained LV dysfunction who have a substantially worse outlook.

Our findings that ACM is associated with an independent OR of 3.06 for LGE presence (95% CI 1.05–8.95), predominantly in the midwall linear pattern, in the context of active alcohol consumption pose the question of whether CMR tissue characterization might help identify patients with a higher risk of fibrosis at an earlier stage who may be less likely to achieve full recovery even after abstinence. Although this hypothesis cannot be tested in the present dataset, it provides a conceptual framework for future longitudinal studies that incorporate serial CMR at baseline, mapping techniques, and follow-up after sustained abstinence to assess for reverse remodeling. The excess of RV dysfunction observed in ACM relative to idiopathic DCM is equally noteworthy from a clinical standpoint. In idiopathic DCM, CMR-assessed RV systolic dysfunction is an independent predictor of transplant-free survival [6], and its systematic characterization has been incorporated into standard CMR reporting and in clinical assessment. Whether the higher prevalence and severity of RV dysfunction in ACM is due to the toxic effects of ethanol and its metabolites or whether it is caused by secondary hemodynamic coupling remains to be determined in independent, prospective studies. Irrespective of mechanism, our data suggest that biventricular CMR assessment in ACM does capture a more complete picture of the myocardial phenotype than LVEF alone, an observation that aligns with the growing body of evidence supporting comprehensive CMR phenotyping in non-ischemic DCM [5,11]. It is important to emphasize, however, that the current study was not designed nor powered to evaluate the prognostic implications of these CMR findings, and any clinical application of these observations would require prospective validation in adequately sized cohorts with extended follow-up. The present data are, therefore, best regarded as hypothesis-generating, providing a rationale for incorporating systematic CMR characterization including biventricular volumetric assessment and LGE into future prospective ACM registries. The midwall linear pattern of LGE, which retains independent statistical significance (25% vs. 8%, *p* = 0.033) and carries well established arrhythmic risk, should be regarded as the primary CMR finding in this cohort rather than the overall LGE prevalence, which is partly driven by the non-specific RV insertion point pattern. When this pattern was excluded, the proportion of patients with non-RV-IP LGE was 9/20 (45%) in ACM patients vs. 29/128 (22.7%) in idiopathic DCM, a difference that was borderline non-significant in our study (*p* = 0.051).

Several limitations must be acknowledged. The retrospective, single-center design and the small ACM sample (n = 20) limit the external validity of these findings and restrict statistical power for outcome analyses. Although all the logistic regression models achieved EPV > 10, the wide confidence intervals of the adjusted OR (1.05–8.95) reflect this limitation and call for discretion in interpretation pending external validation in larger, multicentric studies. Interobserver agreement for LGE pattern classification was not formally quantified in this study with Cohen’s Kappa, as the original CMR image archive was not available for blinded re-reading at the time of revision. This represents a methodological limitation. However, the robustness of the LGE classification is supported by the high level of reader expertise: both readers held more than 5 years of dedicated CMR reporting experience and the ESC Certification in Cardiovascular Magnetic Resonance, a rigorous competency-based accreditation requiring standardized image acquisition, post-processing, and pattern recognition proficiency. Disagreements were resolved by consensus with a third qualified reader. Future prospective studies should report pre-specified interobserver variability assessment with Cohen’s kappa for each LGE subpattern to ensure reproducibility.

The time elapsed between heart failure diagnosis and CMR referral was not systematically recorded in this retrospective registry, and CMR findings, therefore, reflect a heterogeneous mix of disease stages and treatment durations which may limit the generalization of our results. Genetic testing was only performed in 34 cases of DCM (26.6%, 34/128) where the family history suggested a case of a familial DCM, and was not done systematically in all patients in the cohort. Furthermore, data on specific pathogenic variants was not collected in this retrospective registry. This limits the study’s ability to characterize the genetic subgroup and means some of the DCM cases may have genetic causes. However, this does not alter the CMR phenotypic comparison, as both familial and non-familial causes of DCM were pooled in the same group and compared to ACM, consistent with clinical classification of cardiomyopathies. Serial CMR data were not available during follow-up; reverse remodeling after alcohol abstinence and its relationship to baseline LGE burden remain important unanswered questions for future prospective studies.

The test for normality using Shapiro–Wilk confirmed non-normal distributions for most continuous variables in the ACM subgroup, supporting the use of non-parametric methods throughout our statistical analysis. The absence of parametric mapping sequences (native T1 and extracellular volume fraction) was due to the retrospective nature of the study, and it prevents quantification of diffuse interstitial fibrosis, which may be an important component of the alcohol-related myocardial injury not captured by LGE alone. Further studies that aim to confirm these findings should incorporate mapping techniques as part of the standard CMR imaging protocol. All CMR studies were performed on 1.5T systems, ensuring field-strength homogeneity across the cohort. Alcohol intake was quantified by standardized clinical interview rather than biochemical markers, introducing potential recall bias in ACM classification, as many patients tend to underreport their alcohol intake. Future prospective studies should incorporate parametric mapping, systematic abstinence follow-up with serial CMR, and extended outcome data to fully characterize the temporal evolution of the ACM CMR phenotype and to assess its clinical implications regarding risk stratification and prognosis.

## 5. Conclusions

ACM has a distinct CMR phenotype relative to idiopathic DCM, characterized by independently increased odds of LGE presence (OR 3.06, 95% CI 1.05–8.95) with a specific midwall linear pattern, significantly worse RV function, and larger RV volumes, independent of LV systolic function. These findings are exploratory but suggest that ACM may represent a particularly fibrosis-prone phenotype with biventricular involvement within the spectrum of non-ischemic dilated cardiomyopathy. Systematic CMR evaluation in ACM, encompassing both tissue characterization and biventricular functional assessment, may add clinically relevant phenotypic information beyond LVEF. Prospective multicentric studies with extended follow-up, parametric mapping techniques, and serial CMR are needed to determine whether these CMR findings carry independent prognostic value and to formally evaluate their potential role in risk stratification within this patient population.

## Figures and Tables

**Table 1:** Baseline characteristics, CMR parameters, and clinical outcomes.

Variable	ACM (n = 20)	Idiopathic DCM (n = 128)	*p*-Value
**Baseline ** **Characteristics**			
Age, years, median (IQR)	59.5 (52.0–69.8)	65.0 (51.8–73.0)	0.558
Male sex, n (%)	19 (95%)	89 (70%)	0.015 *
Current smoking, n (%)	11 (55%)	31 (24%)	0.007 *
NYHA class ≥ III, n (%)	5 (25%)	26 (20%)	0.570
Prior atrial fibrillation, n (%)	5 (25%)	30 (23%)	1.000
Familial cardiomyopathy, n (%)	1 (5%)	33 (26%)	0.045 *
**CMR—LV Parameters**			
LVEF, %	32.5 (27.5–38.2)	35.0 (26.8–44.0)	0.293
LVEDV/BSA, mL/m^2^	131.5 (112.5–152.2)	120.5 (100.8–149.2)	0.290
LVESV/BSA, mL/m^2^	86.5 (72.0–111.0)	79.5 (59.0–111.0)	0.397
**CMR—RV Parameters**			
RVEF, %	40.5 (35.2–55.0)	52.0 (45.0–59.0)	0.010 *
RVEDV/BSA, mL/m^2^	96.0 (82.8–112.0)	79.0 (68.5–95.0)	0.004 **
RVESV/BSA, mL/m^2^	46.8 (40.8–69.8)	36.0 (28.0–46.2)	0.001 ***
**CMR—Late Gadolinium Enhancement**			
Any LGE, n (%)	14/20 (70%)	51/128 (40%)	0.015 *
Midwall linear, n (%)	5/20 (25%)	10/128 (8%)	0.033 *
Midwall patchy, n (%)	2/20 (10%)	13/128 (10%)	1.000
RV insertion point, n (%)	9/20 (45%)	30/128 (23%)	0.056
Subepicardial, n (%)	0/20 (0%)	7/128 (5%)	0.594
**Follow-up Outcomes (Median 24 Months)**			
MACE, n (%)	7 (35%)	37 (29%)	0.697
HF hospitalization, n (%)	4 (20%)	17 (13%)	0.489
All-cause death, n (%)	2 (10%)	11 (9%)	0.689

ACM, alcoholic cardiomyopathy; BSA, body surface area; DCM, dilated cardiomyopathy; IQR, interquartile range; LVEDV/LVESV, left ventricular end-diastolic/end-systolic volume; LVEF, left ventricular ejection fraction; MACE, composite of all-cause death, heart failure hospitalization, or arrhythmia; RVEDV/RVESV, right ventricular end-diastolic/end-systolic volume; RVEF, right ventricular ejection fraction. Continuous variables compared by Mann–Whitney U test; categorical variables by chi-squared test or Fisher’s exact test as appropriate. * *p* < 0.05; ** *p* < 0.01; *** *p* < 0.001.

## Data Availability

The raw data that support the findings of this study are available from the corresponding author upon reasonable request, subject to applicable data protection regulations (Ley Orgánica 3/2018, de 5 de diciembre, de Protección de Datos Personales).
